# Herpes Zoster Induced Osteomyelitis in the Immunocompromised Patients: A 10-year Multicenter Study

**Published:** 2014-09

**Authors:** Reza Tabrizi, Ali Dehghani Nazhvani, Amir Vahedi, Mehdi Gholami, Raziyeh Zare, Raha Etemadi Parsa

**Affiliations:** a Dept. of Craniomaxillofacial Surgery, School of Dentistry, Shiraz University of Medical Science, Shiraz, Iran.; b Dept. of Oral and Maxillofacial Pathology, School of Dentistry, Shiraz University of Medical Science, Shiraz, Iran.; c Dept. of Pathology, Tabriz University of Medical Science,Tabriz, Iran.; d Dept. of Maxillofacial Surgery, Mashhad University of Medical Science, Mashhad, Iran.; e Postgraduate Student of Oral and Maxillofacial Pathology, Shiraz University of Medical Science, Shiraz, Iran.

**Keywords:** Zona, Osteomyelitis, Mandible, Herpes zoster

## Abstract

**Statement of the Problem: **Alveolar bone necrosis induced by Herpes zoster infection is considered as a rare manifestation of osteomyelitis and few case reports are presented in the literature.

**Purpose: **The aim of this study was to evaluate mandibular osteomyelitis caused by herpes zoster in the immunocompromised patients with histopathologically documented osteomyelitis in the mandible and herpes zoster infection.

**Materials and Method:** 30 patients were recruited in this cross-sectional study. 19 patients were completely edentulous, 4 patients were partially edentulous and 7 with complete dentition. In all cases, specimens were analyzed using a conventional polymerase chain reaction (PCR) test for varicella zoster virus.

**Results: **16 patients underwent dialysis, 9 patients received chemotherapy treatments and 5 patients had transplantation (four kidneys and one liver). Histopathological assessment demonstrated a nonspecific bone necrosis exhibiting an eosinophilic, homogeneous non-vital bone tissue with peripheral resorption surrounded by reactive connective tissue. PCR test was positive in 21 cases.

**Conclusion: **This study demonstrated that the frequency of osteomyelitis induced by herpes zoster could be more than the records provided by previous studies. Histopathological findings might be nonspecific in such patients. PCR test was not positive for all HZ induced osteomyelitis patients**.**

## Introduction

Herpes zoster (HZ), also recognized as Shingles or Zona, is a viral disease presenting with painful skin rash and blisters in a small area on one side of the body, usually with displaying a band. The initial infection with varicella zoster virus (VZV) causes the chickenpox disease in children and young people. The virus can be latent in the nerve cell bodies, less likely in non- neuronal satellite cells of dorsal root, cranial nerve, or autonomic ganglion without producing any clinical symptoms [1-2]. Chickenpox infection may later (long time after initial infection) escape the nerve cell bodies and travel down the nerve axons to cause viral infection of the skin in the region where the infected nerve innervates. The virus could spread from one or more ganglia along the neurons of an involved segment, infecting the innervating dermatome and exhibiting a painful rash [3-4].

Even though the skin rash regularly heals after 2-4 weeks, the nerve pain remains for months or years demonstrating a condition called post-herpetic neuralgia. The incidence rate (per year per 1000 healthy individuals) of herpes zoster ranges from 1.2 to 3.4 in young patients and 3.9–11.8 in patients older than 65 years [5]. Antiviral drug therapy can reduce the severity and duration of herpes zoster if the administration of these drugs is started within 72 hours from the initial presence of the characteristic skin rash and is continued for 7-10 days [5-6]. The thoracolumbar trunk (especially T3 to L3) is most commonly involved in HZ infection(HZI) [7]. HZ may also involve the cranial nerves among which the trigeminal nerve is the most frequently involved (18.5%-22% of total cases). Trigeminal nerve is affected unilaterally and limited to a single division, more often the first (ophthalmic) in HZ patients. Oral manifestations of HZ appear when the second or third division is involved [8].

Other maxillofacial complications of this infection which occur less likely include developmental anomalies such as irregular short roots and missing teeth, facial scarring, periodontitis, calcified and devitalized pulps, periapical lesions, and resorption of the roots [9].

Herpes zoster-induced alveolar bone necrosis is a rare manifestation of this disease and few case reports are available in the literature. This brutal manifestation of the disease is most often noted in immunocompromised and rarely in immunocompetent patients [10]. The aim of this study was to evaluate the mandibular osteomyelitis induced by herpes zoster in the immunocompromised patients.

## Materials and Method

**Figure 1:**
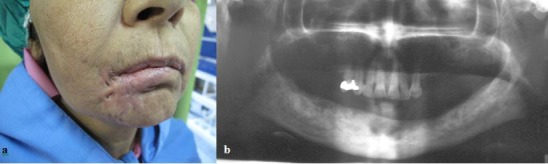
a: Shows scars in the mouth corners of mouth due to herpes zoster  b:  Panoramic radiograph showing a well demarcated sequestrum in the mandibule.

The sample of survey in this cross-sectional study was selected from the population of patients who referred to the pathology departments of three major university hospitals of Iran: Chamran in Shiraz, Imam Reza in Tabriz, and Taleghani in Tehran, from January 1^st^ 2001 to December 31^st^ 2010. These centers are considered as the main referral centers for the patients involved with maxillofacial diseases in their cities. The patients were included in this study if they were immunocompromised and had radiographically and histopathologically confirmed osteomyelitis in their mandible with a concomitant herpes zoster infection. The patients were excluded from the study if they had osteomyelitis caused by odontogenic infection or trauma, a bone metabolism disease, or a history of radiotherapy. The cases were also excluded from the study if the histopathological samples showed a bacterial invasion with odontogenic origin. Dental panoramic radiograph was taken from each patient. The criteria for odontogenic infection included the presence of any radiolucency around the teeth in panoramic radiographs or periapical views, periodontitis, hopeless tooth and odontogenic cysts or tumors, and a positive culture report for odontogenic sources from the infected site. Herpes zoster infection was diagnosed concerning the clinical signs and symptoms. These features included constant pain in the trigeminal nerve (V3), vesiculo-bullous lesions along the trigeminal nerve, thoraco-lumbar dermatome involvement and positive serologic findings that were assessed by employing polymerase chain reaction (PCR) test (Figure 1).

Immunocompromised patients were divided into three groups: 1) Patients who underwent dialysis 2) Patients who received chemotherapy drugs and 3) Patients who underwent transplantation.

In all patients, the acquired specimens were analyzed by employing a conventional PCR test for VZV. Demographic data such as age, gender, and medical history was documented in all patients. The Chemotherapeutic medications provided for the patients included 5-fluorouracil (5-FU), Gemcita-bine (Gemzar®), Methotrexate (MTX), and Cytarabine (Ara-C®).

The main purpose of the current study was the diagnosis of HZ induced osteomyelitis based on the radiological evaluation, clinical assessment and laboratory tests and different treatments of this type of osteomyelitis was not in the scope of this survey.

## Results

30 participants (16 male and 14 female patients), referred to one of the three departments, were immunocompromised and had osteomyelitis in the mandible with herpes zoster infection. The mean age of these patients was 52.6±10.6 years. The most common site of the involvement was the body of the mandible; 12 on the right and 9 on the left side of the mandible. The next most common involved parts were symphysis (in 6 patients) and mandibular angle (in 3 patients) (Table 1).

**Table 1:** Descriptive statistics of the study.

**Study variables **	**Descriptive statistics**		
Age (Years)	52.6 ± 10.6		
Sex	Male16 (53.3%)	Female 14 (46.7%)	
Dentition	ED 19 (63.3%) PE	4 (13.3%) CD 7 (23.3%)	
Medical condition	Dialysis 16 (53.3%)	Chemotherapy 9 (30%)	Transplantation 5 (16.7%)
Site of Involvement (Mandible)	Body 21 (70%)	Symphysis 6 (20%)	Angle 3 (10%)
PCR test	Positive 21 (70%)	Negative 9 (30%)	

ED: Edentulous     PE: Partial edentulous            CD: Complete dentition

**Figure 2:**
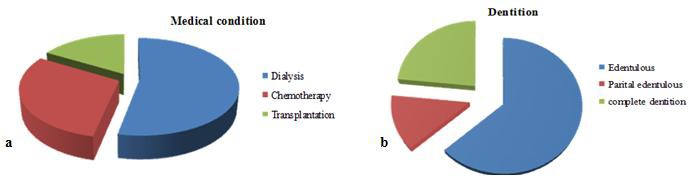
a: Medical condition of the patients  b: Dentition status of the patients.

The results demonstrated that 16 patients underwent dialysis, 9 patients received chemotherapy drugs and 5 patients had transplantation (four kidneys and one liver) (Figure 2a). Evaluation of patients showed 19 patients were completely edentulous, four patients were partial edentulous and seven had a complete dentition (Figure 2b).

**Figure 3:**
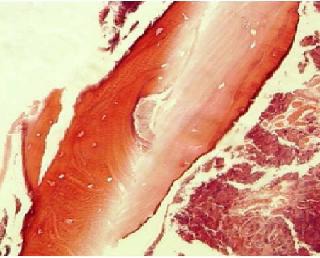
Nonvital bone trabeculae showing loss of osteocytes and surrounding bacterial colonization, 400X magnification.

The mean period of hospitalization for managing the osteomyelitis was 19.1±3.4 days. The surgical intervention for debridement of the mandibular bone was performed in 23 patients. 7 patients were treated by using medication without any surgical intervention. Histopathological evaluation revealed anonspecific bone necrosis in patients, including aneosinophilic, homogeneous non-vital bone tissue with peripheral resorption surrounded by reactive connective tissue. The osteocytic lacunae were empty in the histo pathological specimen (Figure 3). In 6 cases, intertrabecular spaces were filled with necrotic tissue and bacterial colonies.PCR test was positive for VZV in 21

patients. 9 patients had only pain and skin lesion or mucosal lesion without a positive PCR result. None of the patients presented with suppurative osteomyelitis.

## Discussion

Maxillary and mandibular alveolar bone necrosis associated with trigeminal herpes zoster is an uncommon condition. Postherpetic alveolar necrosis and spontaneous tooth exfoliation have been described in 41 cases until 2009 [11]. A review of these previously reported cases shows an age range of 6-85 years with a mean age of 53.3 years [11]. The increasing frequency of HZ infection with age has been speculated concerning the fact that zoster-neutralizing antibodies usually disappear for 40 years after the initial attack of chickenpox infection [7].

To the best of author’s knowledge, the current study is the first long-term study enrolled on the immunocompromised patients. Three main university hospitals in the North west, capital, and the South of Iran were selected who have thousands of referral patients needing dialysis or chemotherapy and many of them suffer from oral problems. This study covered a broad geographical area, therefore identifying the osteomyelitis cases without odontogenic sources could be unavoidable during 10 years; this rationale can justify the period of time designated in this study. The current study demonstrated that the incidence of osteomyelitis associated with HZ was more than earlier expectations. Osteomyelitis occurred more commonly in patients who underwent dialysis and had HZ infection. Dialysis patients were more susceptible to viral infection because of possible contamination during dialysis.

The majority of patients were edentulous which reveals that odontogenic sources could not be considered as an important factor. In our study, no patients had suppurative osteomyelitis, based on which it can be explicated that the studied patients were immunosuppressed, therefore their immunologic system was too weak to react and form the pus [8]. PCR has a high specificity to VZV [3]. However, in our study the results of the PCR test in 9 patients were negative and they only showed skin lesions of Zona. Osteomyelitis is a rare complication occurring after varicella infection [12]. A study showed that post -HZ osteonecrosis has a slight predilection for mandible (18 patients) rather than the maxilla (13 patients), and more teeth lost was observed in mandible (44 teeth) compared to the maxilla (31teeth). Of the teeth lost, 64 were anterior teeth and 61 were posterior teeth; one study did not specify the location of the lost teeth [13].

Mintz and Anvavi reported a case of maxillary osteomyelitis with spontaneous tooth exfoliation after infection with herpes zoster in a 50-year-old man without presenting any immunological problem [14].

Jain et al. introduced a case of osteonecrosis complicated with mandibular pathologic fracture following herpes zoster infection, without demonstrating any other predisposing factors [11]. Reports of spontaneous tooth exfoliation and jaw osteonecrosis following herpes zoster infection in the distribution of the trigeminal nerve are extremely infrequent with only 39 cases being reported in the literature 15. Meer et al. reported an additional case of mandibular osteomyelitis and spontaneous tooth exfoliation subsequent to herpes zoster infection, which occurred in the left mandible of a 70-year-old diabetic male individual with cytomegalo virus (CMV) co-infection [15]. Extensive osteonecrosis and exfoliation of teeth in the area innervated by HZ –affected nerve, has been reported after HZ infection [9].

15 patients had underlying disease, including hematologic neoplasm, such as Hodgkin's disease, chronic hepatitis, diabetes mellitus, acquired immunodeficiency syndrome (AIDS), tuberculosis and i*mmunosuppression *due to kidney transplantation. Herpes zoster infection was severe in all cases and the involved sites included the maxillary nerve in eleven, mandibular nerve in eighteen, ophthalmic and maxillary nerve in one, and maxillary and mandibular nerve in two patients. Maxillary and mandibular alveolar bone necrosis appeared 9–150 days (with a mean of 30 days) after the onset of herpes zoster. Thirteen patients required extraction a few teeth, while the others required extensive tooth extraction. The teeth in the affected segment spontaneously exfoliated 9-150 days after infection with HZ in some cases [1-9].

The pathogenesis of alveolar necrosis is still controversial, although two hypotheses have been postulated. One possible elucidation could be the presence of ischemia. The vasculitis induced by the VZV may lead to necrosis of the periodontal tissue and alveolar bone [8, 16]. An alternative rationale is that the inflammatory edema of the alveolar nerve might compress the alveolar artery in the narrow maxillary or mandibular canal. This process could result in ischemia and consequent necrosis of the periodontal tissue and alveolar bone [3, 7], whilst pre-existing pulpal and periodontal infection may also contribute to this mechanism [1].

Another explanation could be the bacterial invasion through the blisters or through the vulnerable areas such the area of acantholysis of the mucosa in the affected region caused by VZV. In an immunocompromised patient, a long-lasting mucosal ulcer provides a perfect entry for actinomycete and staphylococcus microorganisms to invade, reside and form osteomyelitis [15]. In literature review, VZV-induced secondary osteomyelitis has not been reported in healthy individuals, moreover, stress has not been considered as an etiological factor [15].

Currently, numerous laboratory diagnostic methods have been developed for the diagnosis of HZ infection, including dot-blot hybridization, PCR and direct staining of cytological smears with fluorescent monoclonal antibodies for VZV. Along with these, histopathological examination of the necrotizing alveolar bone has been proposed [10].

## Conclusion

This study demonstrated that the frequency of osteomyelitis caused by HZ could probably be more than the reported previous studies. Histopathological findings might be nonspecific in such patients.PCR test was not positive for all HZ induced osteomyelitis patients.
